# Pediatric Acute Lymphoblastic Leukemia Patients Exhibit Distinctive Alterations in the Gut Microbiota

**DOI:** 10.3389/fcimb.2020.558799

**Published:** 2020-10-16

**Authors:** Xiaoming Liu, Yao Zou, Min Ruan, Lixian Chang, Xiaojuan Chen, Shuchun Wang, Wenyu Yang, Li Zhang, Ye Guo, Yumei Chen, Yingchi Zhang, Hongrui He, Yu Gan, Kejian Wang, Xiaofan Zhu

**Affiliations:** ^1^State Key Laboratory of Experimental Hematology, National Clinical Research Center for Blood Diseases, Division of Pediatric Blood Diseases Center, Institute of Hematology & Blood Diseases Hospital, Chinese Academy of Medical Sciences & Peking Union Medical College, Tianjin, China; ^2^College of Biological Science and Engineering, Fuzhou University, Fuzhou, China; ^3^Lin He's Academician Workstation of New Medicine and Clinical Translation at The Third Affiliated Hospital, Guangzhou Medical University, Guangzhou, China

**Keywords:** interleukin-10, quantitative PCR, 16S rRNA quantitative microarray, gut microbiota, acute lymphoblastic leukemia (ALL)

## Abstract

Previous studies have shown that gut microbiota can affect human immune system in many ways. Our aim was to investigate quantitative differences in fecal bacterial compositions of childhood acute lymphoblastic leukemia (ALL) patients compared to those of healthy children, so as to identify individual bacterial species that are related to the etiology of ALL. We recruited 81 subjects, including 58 patients with ALL and 23 healthy controls. Fecal samples were collected and examined by 16S rRNA quantitative arrays and bioinformatics analysis. Both Principal Coordinates Analysis (PCoA) and Non-metric Multidimensional scaling (NMDS) demonstrated that the microbial composition of ALL patients deviated from the tight cluster of healthy controls. Multiple bacterial species exhibited significant changes (e.g., *Roseburia faecis, Edwardsiella tarda*, and *Fusobacterium naviforme*) in the ALL samples. Some of the differentially abundant taxa were correlated with the level of interleukin-10. The ALL cases could be efficiently distinguished from healthy controls by the random forest model based on differential species (area under ROC curve = 0.843). Taken together, the composition of gut microbiota differed from healthy controls to pediatric ALL patients. Our study identified a series of ALL-related species in the gut microbiota, providing a new direction for future studies aiming to understand the host-gut microbiota interplay in ALL pathogenesis.

## Introduction

Acute lymphoblastic leukemia (ALL) is the cancer affecting the lymphoid line of blood cells, which is characterized by dramatically increasing numbers of immature lymphocytes. The treatment of pediatric ALL is one of the greatest successes of modern medicine (Schotte et al., 2011), since certain subtypes of ALL (e.g., TEL-AML1-translocated ALL) have a favorable 5-years survival over 85% (Meijerink et al., 2009). However, MLL-rearranged, BCR-ABL-positive and T cell ALL still have an unfavorable 5-years survival (Pieters et al., 2007).

It has been widely reported that gut microbiota is intricately connected with immune system and provides crucial signals for immunity function (Spencer et al., 2019). Gut microorganisms can produce a variety of metabolites from fermentation in the colon. The major metabolic end-products are short-chain fatty acids, which may influence the expansion of haematopoietic cell lineages by interacting with G protein-coupled receptors (GPCRs) and histone deacetylases (HDACs) (Rooks and Garrett, 2016). In particular, a number of clinical studies demonstrated that gut microbiota is closely related to ALL. Chua et al. reported microbial dysbiosis and inflammation/immune dysregulation in adult survivors of childhood ALL (Chua et al., 2017). Hakim et al. found that in pediatric ALL patients, the composition of gut microbiota before chemotherapy initiation can predict the incidence of infection and febrile neutropenia in subsequent phases of treatment (Hakim et al., 2018). Rajagopala et al. assessed the gut microbiota profiles in pediatric and adolescent ALL patients during the course of chemotherapy (Rajagopala et al., 2016). However, there is still very limited evidence clearly showing the abnormalities of the gut microbiota in ALL patients as compared to healthy controls.

Utilizing a novel high-throughput microarray technology, it has been practical to quantify various bacteria taxa a given sample, without the need for conventional culture-based techniques. In this study, we applied 16S rRNA quantitative microarrays (Zheng et al., 1999) to investigating potential alterations in the gut microbiota of pediatric patients with ALL, as compared to that of controls. Furthermore, we studied whether the microbiota profiles were related to immune parameters in ALL patients.

## Materials and Methods

### Study Design and Sample Collection

We recruited 70 newly occurring children with acute lymphoblastic leukemia (ALL) at the Division of Pediatric Blood Diseases Center, Institute of Hematology & Blood Diseases Hospital, Chinese Academy of Medical Sciences & Peking Union Medical College, State Key Laboratory of Experimental Hematology, National Clinical Research Center for Blood Diseases from November 2018 to March 2019. At the same time, 35 healthy volunteer children (non-ALL controls, NC) from the same region were also recruited in the study. They came from local kindergartens and primary and secondary schools.

All the acute lymphoblastic leukemia (ALL) patients eligible for this study were diagnosed with ALL according to the morphologic, cytochemical, and immunophenotypic criteria (Swerdlow et al., 2017). Patients who were mature B-ALL, mixed phenotype leukemia, with secondary tumors, definite changes in CML, secondary to immunodeficiency disease, those using glucocorticoids or any chemotherapy or radiotherapy within 3 months were excluded. The healthy controls exhibited no disease symptoms. Each participant was assessed using the Rome III Criteria to exclude irritable bowel syndrome (IBS) (Koloski et al., 2015), which has been shown to influence gut microbiota (Kassinen et al., 2007). Individuals with severe chronic diseases (eg, diabetes, heart failure, cirrhosis, or autoimmune diseases) were excluded. Individuals currently taking antibiotics, probiotic supplements or NSAIDs within the three months prior to sample collection were also excluded.

A total of 58 ALL patients (34 [58.6%] male; mean [standard deviation, SD] age 7.2 [3.6] years) and 23 healthy volunteer children (11 [47.8%] male, mean [SD] age 7.6 [3.6] years) were included in the final analysis. Twelve children with ALL and 12 healthy volunteer children were excluded because of their currently taking antibiotics, probiotic supplements or NSAIDs within the 3 months.

Each participant was informed of the purpose of this study, and all measurements and questionnaires were voluntary. All enrolled subjects provided written informed consent. This study protocol was approved by the Research Ethics Committee, Institute of Hematology & Blood Diseases Hospital, Chinese Academy of Medical Sciences & Peking Union Medical College, State Key Laboratory of Experimental Hematology, National Clinical Research Center for Blood Diseases, Tianjin, China.

All children with ALL were given stools when the disease was diagnosed but no treatment was started. All healthy volunteer children retained their stools when they joined the study. The fresh stool samples were collected by using an institution approved protocol. Stool specimens were collected and saved in the sampling tubes with preservative solution. The tubes and preservative solution were provided by Halgen Ltd (China).

### Human Gut Bacterial Microarrays

The human gut bacterial microarrays used in this study were designed and manufactured by Halgen Ltd. using its proprietary oligo array technology. The arrays contain 4,751 high-specificity and high-sensitivity oligo probes representing non-redundant 1,485 bacterial species belonging to 418 genera. The arrays cover over 95% of culturable gut microbial species found in different population. Each oligo probe was printed in triplicate on the array. A panel of 28 reference species were used to validate the specificity of the probes and hybridization condition by Halgen Ltd. (Bradley and Cai, 2000; Bradley et al., 2005; Gibbs et al., 2005).

### DNA Extraction and Labeling

Bacterial DNA was extracted from the stool samples using the Stool DNA Extraction Kit (Halgen, Ltd.) and following the protocol described in the product instruction manual. A universal primer pair was used to amplify the DNA the V1-V9 regions of the 16S rRNA gene. Approximately 20–30 ng of the extracted DNA was used in a 50 ul PCR reaction using the following cycling conditions: 94°C for 3 min for an initial denaturing step followed by 94°C for 30s, 55°C for 30s, 72°C for 60s for a total of 30 cycles followed by a final extension step of 72°C for 3 min. Agarose gel electrophoresis was run to check the success of PCR amplification. The PCR products were directly labeled without purification using a DNA labeled kit provided by Halgen Ltd. and processed for array hybridization according to the product instruction manual.

### Microarray Hybridization

Probes were selected from all the variable regions of bacterial 16S rRNA of bacteria. Each probe was designed to be about 40 bp in length. The arrays were prepared by Halgen Ltd. using its proprietary technology. The hybridization mixes typically contained 500 ng of Cy5-labeled test sample DNA mixed with 50 ng of a Cy3-labeled reference pool which serve to light up all the spots for accurate identification of spots and signal quantitation. The Cy3- and Cy5-labeled samples and hybridization buffer (Halgen Ltd.) were mixed in a final volume of 150 μl, heated to 100°C for 5 min, and cooled on ice for 5 min. All of these were put into a hybridization box and then hybridized in a hybridization oven with intermittent invention (Halgen Ltd.) for 3.5 h at 37°C. Slides were washed in 2 × SSC, 0.25% Triton X-100, 0.25% SDS, 1X Dye Protector (Halgen Ltd.) for 15 min at 63°C, then rinsed in 1X Dye Protector till the slides clear of water droplets after immediate withdraw from the solution. Slides were scanned immediately using a dual-channel scanner (Genepix 4000B). The microarray raw data have been deposited into CNGB Sequence Archive (Guo et al., 2020) of CNGBdb (Chen et al., 2020) with accession number CNP0001267.

### Data Analysis

The program first calculated the mean intensities of the triplicated spots of each species for the Cy5 and Cy3 channels. The mean Cy5/Cy3 intensity ratio was calculated from the mean intensity of the respective channels. The species were ranked by their mean signal intensity from which the background fluorescent signal was calculated from the mean of the mean intensity of the lower 30% of the species. For a species to be judged as positive detection, its mean signal intensity must be 5-fold higher above the background. We further filtered out the first-pass positive species by checking their Cy5/Cy3 ratio rankings. The baseline Cy5/Cy3 ratio was calculated from ranking the Cy5/Cy3 ratios from all the spots. The mean of the lower half of the Cy5/Cy3 rankings was taken for the baseline Cy5/Cy3 ratio. For the second-passed species only those with a Cy5/Cy3 ratio >5-fold of the baseline Cy5/Cy3 value were positive. To calculate the relative abundance of the positive diatom species, we normalize the Cy5/Cy3 ratio of all the third-passed positive species and the percentage of each species is presented as the relative abundance value.

Alpha-diversity was calculated using and QIIME software (Kuczynski et al., 2011) with default parameters. The differences of alpha-diversities between groups were calculated by Wilcoxon rank-sum test. PCoA and NMDS analyses were performed by QIIME modules and visualized by R packages (version 3.5.2). Linear Discriminant Analysis (LDA) Effect Size (LEfSe) (Segata et al., 2011) analysis was performed to analyze difference of bacterial species between groups. The *p*-value for each species were calculated by Kruskal-Wallis test and Wilcoxon test. Unsupervised random forest classification and receiver operating characteristic curve (ROC curve) proportional hazards statistics were also performed using R. To reduce the impact of overfitting in random forest, cross validation was performed following leave-one-out procedure.

### Quantitative Real-Time PCR

The qPCR primers were designed for species-specific amplification 16S rRNA gene of *Roseburia faecis* (Forward 5′-GAACGAAGCACTCTATTTGATTTTCT-3′; Reverse 5′-TCACACCGGATCATGCGAT-3′), *Edwardsiella tarda* (Forward 5′-GAGGAAGGTGTGCGTGT-3′; Reverse 5′-CGGTGCTTCTTCTGTAGGT-3′), *Fusobacterium naviforme* (Forward 5′-CGCGTCTAGGTGGTTATGT-3′; Reverse 5′-TCTGTCCAGTAAGCTGGCT-3′). The specificity was confirmed by Sanger sequencing of amplicons from partial samples as well as melting curve analysis of all amplicons following SYBR Green qPCR amplification. For normalization of DNA input and total bacteria content among samples, a universal primer pair targeting 16S rRNA consensus sequence was used as a reference (Clifford et al., 2012). Template genomic DNA of 50 ng input and each PCR primer pair (0.4 μM for 16S specific PCR and 0.2 μM for 16S universal PCR) were added to SYBR Green qPCR mix with ROX (BeyoFast TM, China). Real-time PCR reactions were carried out in an ABI 7500 instrument under thermal conditions of 95°C for 3 min, followed by 45 cycles of 95°C for 20s, 56°C for 20s, and 72°C for 30s. The fluorescence was recorded during the 72°C extension step. Melting curve analysis was run following amplification. The threshold cycle (Ct) of each sample for each target was recorded as average value of duplicates. The relative abundance of each bacterium is expressed as 2^∧^ [Ct (16S universal PCR)-Ct (16S specific PCR)] multiply 10,000.

### Cytokine Level Detection

Venous blood was collected from each patient under fasting conditions. Serum IL-1β, IL-2R, IL-6, IL-8, IL-10, and TNF-a were determined at ALL diagnosis (before any treatment) using the chemiluminescence (CL) assay (SIEMENS IMMULITE1000) with the kit purchased from Siemens Healthcare Diagnostics Products Limited. All the procedures were carried out at least in triplicate according to the manufacturer's instructions (Wang and Yang, 2017).

## Results

### Characteristics of Study Subjects

The demographic characteristics of ALL group and non-ALL control (NC) group were summarized in Table 1. No differences in age, gender, birth order, birth weight, feeding pattern, diet, constipation, or BMI were detected between the two groups (Table 1). All children with ALL were not given any chemotherapy drugs until the first stool was taken.

**Table 1:** Characteristics of study subjects.

	**ALL group**	**Control group**	***P*-value**
	**(*n* = 58)**	**(*n* = 23)**	
Age [year, median (range)]	7.2 (3.0–15.0)	7.6 (3.0–16.0)	0.588
Gender			0.896
Male (*n*, %)	34 (58.6)	11 (47.8)	
Female (*n*, %)	24 (41.4)	12 (52.2)	
Birth order			0.129
First born (*n*, %)	37 (63.8)	18 (78.3)	
Second child (*n*, %)	18 (31.0)	4 (17.4)	
Third and upper (*n*, %)	3 (5.2)	1 (4.3)	
Modes of delivery			0.600
Spontaneous delivery	34 (58.6)	12 (52.3)	
Cesarean section	24 (43.4)	11 (47.8)	
Birth weight			0.433
≤ 3 kg (*n*, %)	18 (31.0)	5 (21.7)	
>3 kg (*n*, %)	40 (69.0)	18 (78.2)	
Feeding patterns			0.768
Breastfeeding (*n*, %)	46 (79.3)	17 (73.9)	
Non-breastfeeding (*n*, %)	12 (20.7)	6 (26.1)	
Diet			0.344
Not picky eaters (*n*, %)	44 (75.9)	15 (65.2)	
Meat-based (*n*, %)	12 (20.7)	7 (30.4)	
Vegetarian-based (*n*, %)	2 (3.4)	1 (4.3)	
Constipation (*n*, %)	9 (15.5)	4 (17.4)	0.837
BMI [kg/m^2^, median (range)]	27.8 (21.4–38.8)	25.9 (21.5–32.0)	0.104

### Overall Differences Between ALL and NC

**Figure 1:**
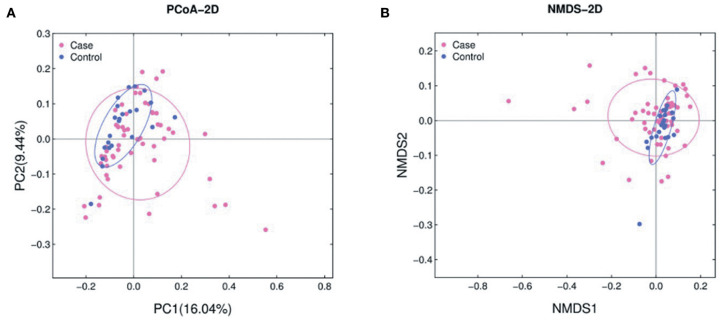
PCoA **(A)** and NMDS **(B)** analyses of β-diversity based on the Bray-Curtis dissimilarity. The stress value of NMDS is 0.17. ALL and NC subjects are colored in pink and blue, respectively.

The abundance of various bacterial taxa for each fecal sample was examined by 16S rRNA quantitative microarrays (see Materials and Methods). Analysis of α-diversity (i.e., Chao, ACE, Shannon, and Simpson indices) showed no significant differences between ALL and HC subjects (*P* > 0.05, Supplementary Figure 1). On the other hand, β-diversity was calculated using Bray-Curtis distance and visualized in both Principal Coordinate Analysis (PCoA) and Non-metric Multidimensional Scaling (NMDS) plots. The results demonstrated that the gut microbiota of ALL clustered apart from that of HC (Figure 1 and Supplementary Figure 2, PERMANOVA *P* < 0.05 for both PCoA and NMDS).

### Bacterial Species With Differential Abundance

**Figure 2:**
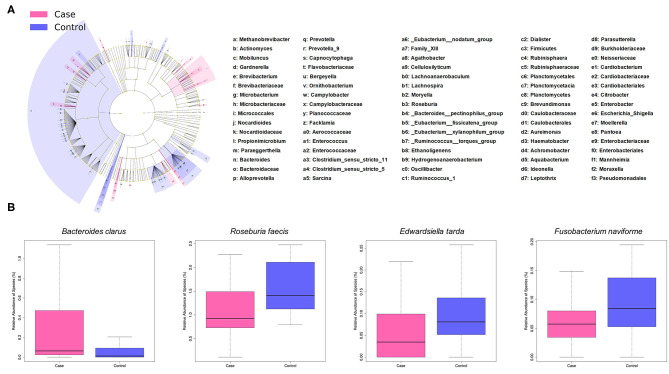
Taxonomic differences of gut microbiota between ALL and control groups. **(A)** Cladogram showing taxa enriched in ALL group (pink) and control group (blue). **(B)** Boxplot showing typical species with significant distinction between two groups.

**Figure 3:**
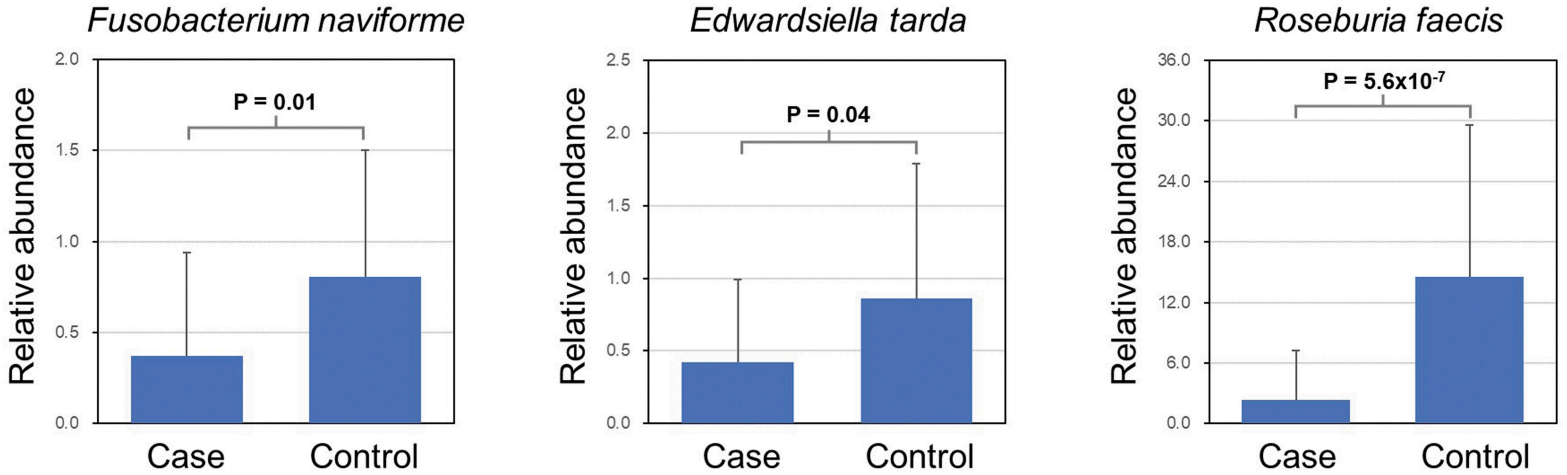
Validation of differential abundance by quantitative real-time PCR.

LEfSe analysis revealed a number of bacterial taxa with increased or decreased relative abundance in ALL than in NC group (Figure 2A, Supplementary Table 1). A series of species were significantly enriched in ALL patients, such as *Bacteroides clarus*. On the other hand, some species exhibited higher relative abundance in the NC group, such as *Roseburia faecis, Edwardsiella tarda*, and *Fusobacterium naviforme* (Figure 2B). These results were further confirmed by quantitative real-time PCR (Figure 3, see Materials and Methods).

**Figure 4:**
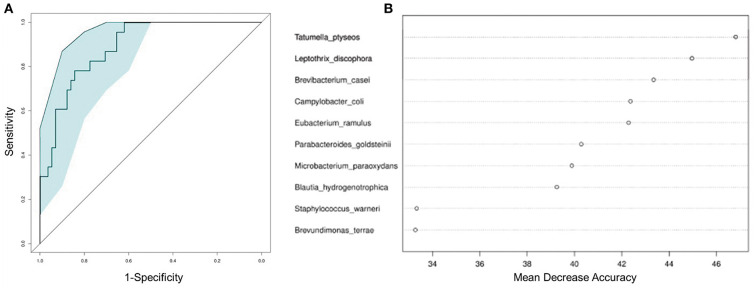
Classification of ALL status by abundance of bacterial species. **(A)** Classification performance of the random forest model was assessed by area under the ROC curve. **(B)** The top 10 discriminant species in the model.

To test whether the microbiota features can identify ALL status, we trained a random forest (RF) model with bacterial abundance data of all the ALL and NC subjects. The efficacy of RF was evaluated following the leave-one-out cross-validation (LOOCV) approach, in which training was based on the whole sample set but left only one sample for the testing purpose, and then such procedure iterated for each sample. The discriminatory power of LOOCV was measured by a receiver operating characteristic (ROC) curve. Given the area under the ROC curve (AUC), we found that the model based on species data outperformed those based on genus, family, and order data (Supplementary Figure 3). ALL patients were efficiently identified by the species-level model with AUC = 0.843 (95% CI: 0.744–0.942, Figure 4A). Most predictive power was derived from the abundance of several species, such as *Tatumella ptyseos, Leptothrix discophora*, and *Brevibacterium casei* (Figure 4B).

### Interrelations of Gut Microbiota and Immune Function in ALL

**Figure 5:**
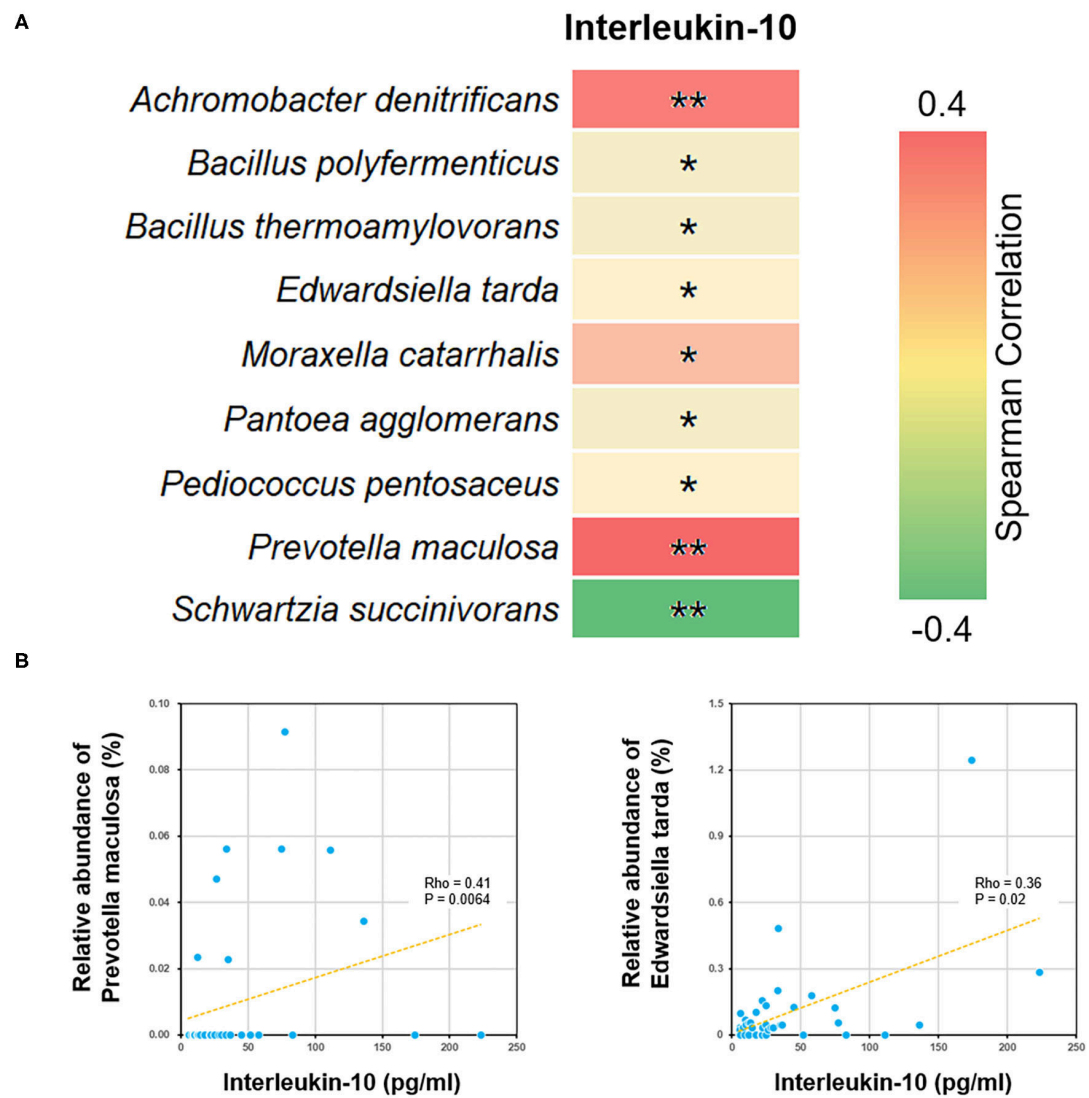
Correlations between bacterial species and blood interleukin-10 level. **(A)** The heat map showed all the species with significant Spearman correlation coefficients in the ALL group. *P < 0.05; **P < 0.01. **(B)** The scatter plots showing IL-10 level and the relative abundance of *Edwardsiella tarda* and *Prevotella maculosa*.

We further performed a Spearman correlation analysis, in order to evaluate the potential interrelation between bacterial species and immune functions. Measured by *P*-value, a number of species were found to be positively or negatively associated with blood test parameters (Figure 5). For example, the level of interleukin-10 was positively correlated to several species, such as *Edwardsiella tarda* and *Prevotella maculosa*. Such correlations implicated the influence of gut microbiome on immune system (see Discussion below).

## Discussion

The correlation between ALL and gut microbiota has been studied from various perspectives, including the long-term effect of chemotherapy (Chua et al., 2017), adverse events during the course of chemotherapy (Hakim et al., 2018), and the gut microbiota signatures for ALL patients of different ages (Rajagopala et al., 2016). However, there remained very limited metagenomic data directly displaying the differences between ALL patients and healthy subject. Our study filled the blanks with a comparison between 58 ALL patients and 23 non-ALL controls, providing the most straightforward answer so far.

The present study provided the first evidence that childhood ALL patients exhibit gut microbiota compositions significantly different from those of healthy subject. Although the α-diversity was even in two groups, the microbial dysbiosis of ALL group was characterized by the differential abundance of multiple bacterial taxa. Both PCoA and NMDS analyses on microbial composition data suggested that healthy controls spontaneously aggregated into a compact cluster, while the ALL patients scattered away from that cluster. In addition, our results showed that the abundance of certain species can used to build up random forest model and distinguish ALL patients from healthy controls. These findings indicated that an ALL-specific signature could be extracted from metagenomic data of a large sample, which may serve as effective biomarkers to help early prevention and clinical management of ALL.

Our results indicated no significant difference in α-diversity between ALL and HC subjects, while other microbiome studies on with only ALL subjects (i.e., without healthy controls) reported changes in α-diversity during or after treatment (Chua et al., 2017; Lee et al., 2019). This is probably because the abundance of some but not all taxa are altered in the early onset of ALL, while drastic changes in most taxa occur during the course of chemotherapy and remission.

By analyzing differential bacterial species between ALL and NC groups, we found that the relative abundance of *Edwardsiella tarda* and *Prevotella maculosa* were reduced among the childhood ALL patients and positively correlated with the level of interleukin-10 (IL-10). *Edwardsiella* (Li et al., 2019) and *Prevotella* (Kostic et al., 2013) were both reported to be involved in immune functions. Their correlations with IL-10 is of particular interest, since several polymorphisms and splicing variants of IL-10 gene has been identified as predictive biomarkers for ALL (Wu et al., 2005; Lo et al., 2016; Ghufran et al., 2019). Moreover, the neonatal level of IL-10 was found to be significantly lower among childhood ALL cases than controls. Here we observed the co-occurrence of declined IL-10 level and decrease in abuandance of *Edwardsiella tarda* and *Prevotella maculosa* suggested that dysbiosis may profoundly influence the pathogenesis of ALL through affecting certain cytokine of immune system (Schirmer et al., 2016).

In this study, the *Fusobacterium naviforme* species exhibited higher abundance in the NC group. Such finding is in line with previous research suggesting that *Fusobacteria* was less abundant in the oral microbiota of ALL patients compared to that of healthy children (Wang et al., 2014). Given the profound relationship between the gut and oral microbiome (Rooks and Garrett, 2016), further study could aim to investigate whether oral bacteria affect the gut microbiota of childhood ALL patients.

Unlike most previous research on gut microbiota, the presents study applied microarray rather than sequencing platform. The major advantage of 16S rRNA quantitative microarray is quantification of various bacterial taxa at species level. In particular, the differential abundance identified by microarray could be effectively validated by qPCR experiments. Due to the limited length of sequencing reads, conventional 16S rRNA sequencing can only cover a relatively short region (e.g., V2-V3) and provide abundance data at genus level or above. The innovative microarray technique enabled us to make deeper phylogenetic analysis of gut microbiota, which helped to better explain the gut dysbiosis in ALL and discriminate between ALL cases and normal subjects (Tu et al., 2017).

However, certain limitations of the present study should also be noticed. Like most studies on gut microbiota, our analysis was mainly based on grouping (ALL vs. NC), but it did not adjust for potential confounders that might contribute to dysbiosis, such as age, gender, diet, and BMI. In addition, since all the subjects were recruited from the same hospital, potential regional variations of gut microbiota could not be assessed. Therefore, multi-center clinical trials or animal models would be required to further corroborate our findings.

In conclusion, the present study provided clear-cut evidence on the abnormalities of gut microbiota in ALL patients as compared to healthy controls. Our results can guide the development of microbiota biomarkers for early prediction of ALL risks and probiotics regimens that alleviate gut dysbiosis in ALL.

## Data Availability Statement

The microarray raw data have been deposited into CNGB Sequence Archive of CNGBdb with accession number CNP0001267.

## Ethics Statement

The studies involving human participants were reviewed and approved by Ethics Committee of Blood Diseases Hospital, Chinese Academy of Medical Sciences. Written informed consent to participate in this study was provided by the participants' legal guardian/next of kin.

## Author Contributions

XZ and KW contributed to the design and monitor of the study. XL, YZo, MR, LC, XC, SW, WY, LZ, YGu, and YC enrolled all the patients and conducted the clinical experiments. XL collected clinical data and wrote the manuscript. HH and YZh did the detection of gut microbiota. KW and XL analyzed the data. XL did the rest of the whole experiments. YGa proofread part of the manuscript. All authors discussed the results and contributed to the final manuscript.

## Conflict of Interest

The authors declare that the research was conducted in the absence of any commercial or financial relationships that could be construed as a potential conflict of interest.

## Supplementary Material

Supplementary Figure 1 Comparison of α-diversity between the gut microbiota of ALL cases and healthy controls. Four indexes were calculated to represent the α-diversity (i.e., Chao index, Ace index, Shannon's diversity index, and Simpson's diversity index).

Supplementary Figure 2 PCoA plots with regard to various stratifying clinical factors.

Supplementary Figure 3 The random forest model based on species data outperformed those based on other taxonomy levels.

Supplementary Table 1 Species showing significant differential abundance between ALL and control groups.
