# Supplementary figures and images for: Pediatric Acute Lymphoblastic Leukemia Patients Exhibit Distinctive Alterations in the Gut Microbiota
Source: Front Cell Infect Microbiol. 2020 Oct 16;10:558799. doi: 10.3389/fcimb.2020.558799 (PMC7596659; doi:10.3389/fcimb.2020.558799)

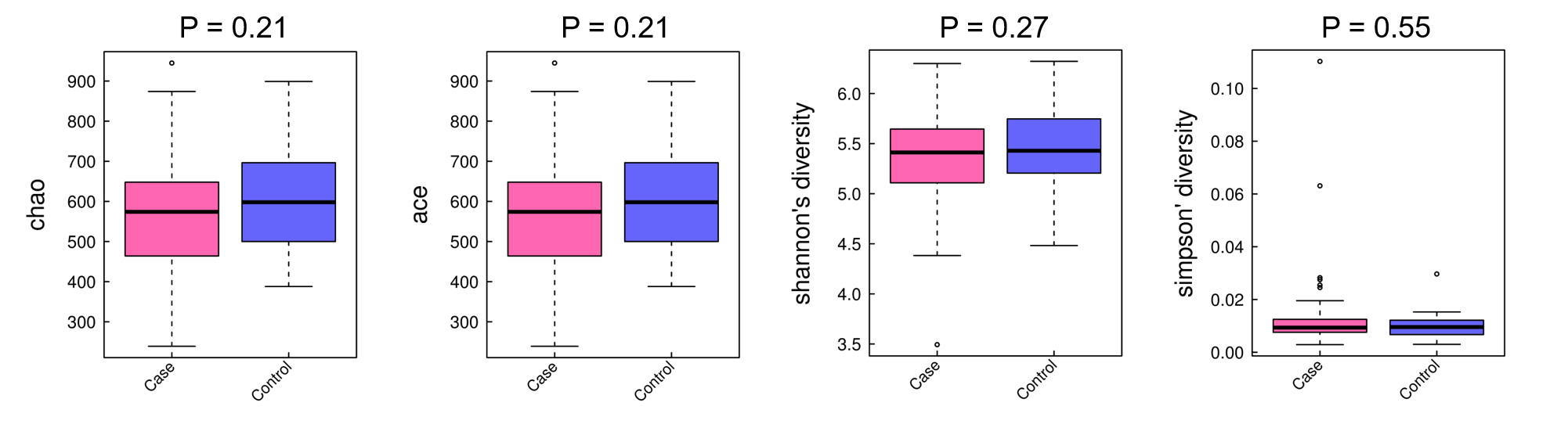

Supplement: Supplementary Figure 1 — Comparison of α-diversity between the gut microbiota of ALL cases and healthy controls. Four indexes were calculated to represent the α-diversity (i.e., Chao index, Ace index, Shannon's diversity index, and Simpson's diversity index). [file Image_1.TIF]

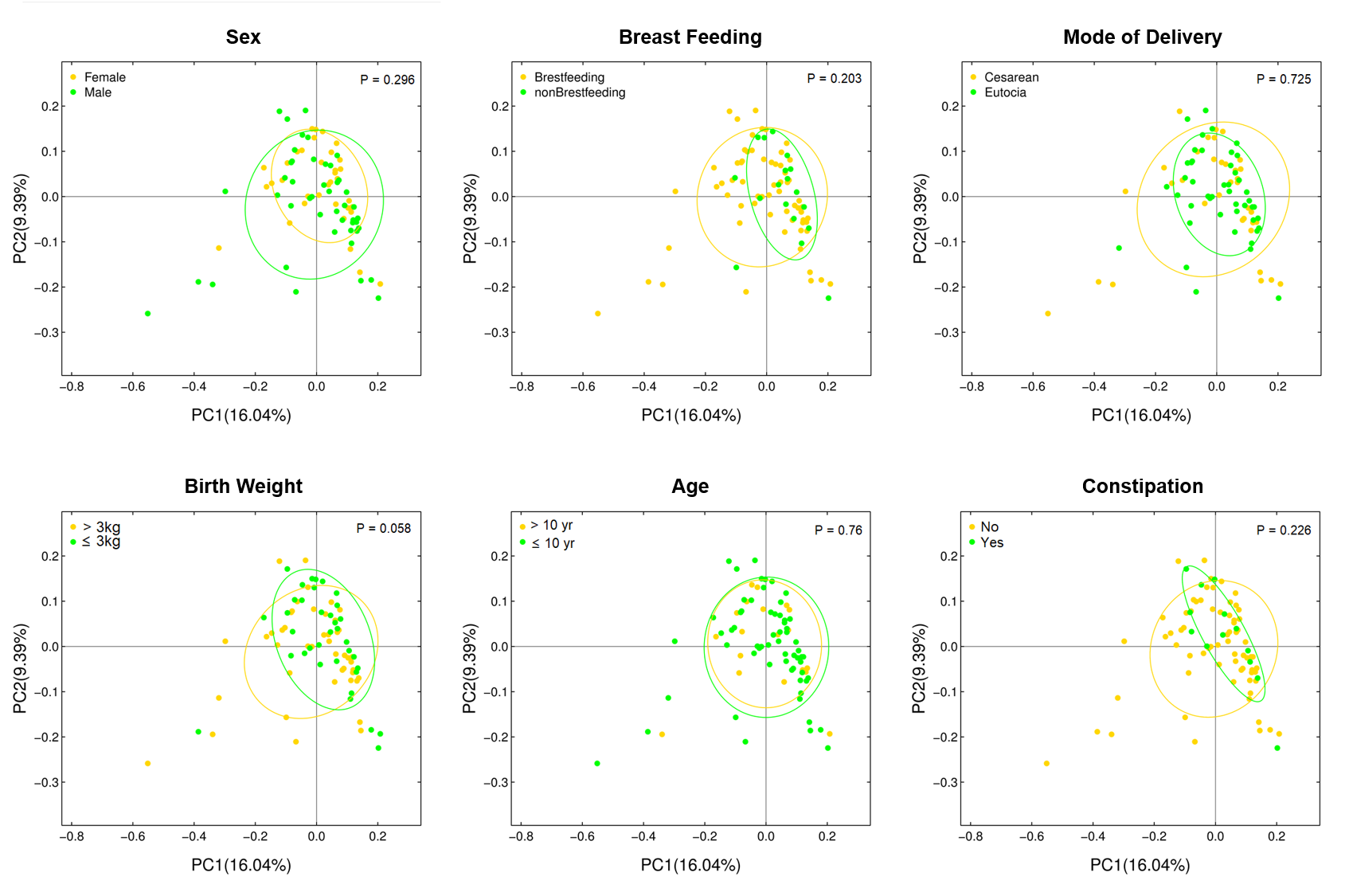

Supplement: Supplementary Figure 2 — PCoA plots with regard to various stratifying clinical factors. [file Image_2.TIF]

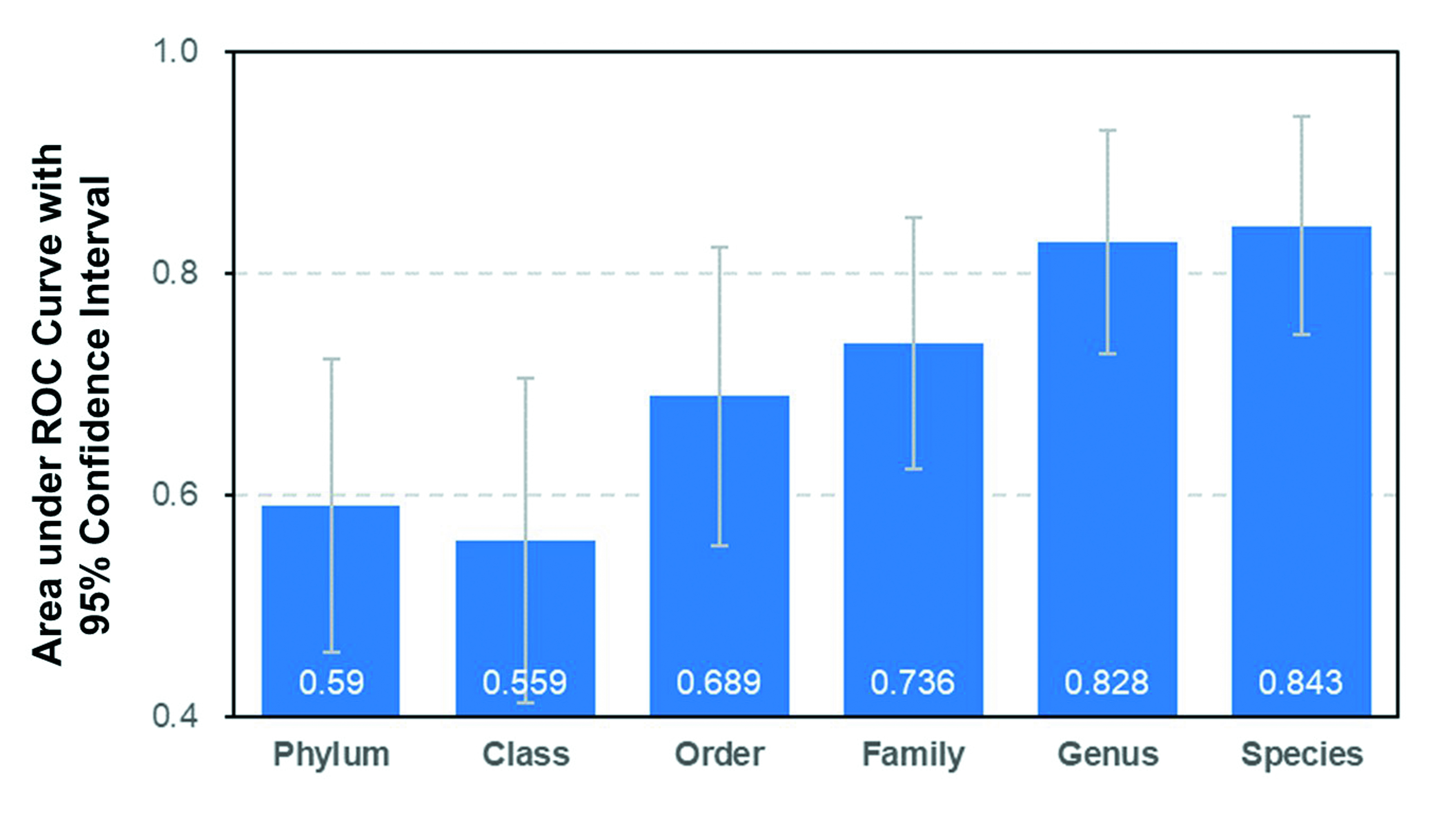

Supplement: Supplementary Figure 3 — The random forest model based on species data outperformed those based on other taxonomy levels. [file Image_3.JPEG]
