# Supplementary material for: Pediatric Acute Lymphoblastic Leukemia Patients Exhibit Distinctive Alterations in the Gut Microbiota
Source: Front Cell Infect Microbiol. 2020 Oct 16;10:558799. doi: 10.3389/fcimb.2020.558799 (PMC7596659; doi:10.3389/fcimb.2020.558799)
Supplement: Supplementary Table 1 — Species showing significant differential abundance between ALL and control groups. [file Table_1.docx]

**Table S1.** Species showing significant differential abundance between ALL and control groups.

| **Species** | **Change in ALL Group** | **Average Abundance in Case Group (%)** | **Average Abundance in Control Group (%)** | **P-value** |
| --- | --- | --- | --- | --- |
| Moraxella catarrhalis | Decreased | 0.096720511 | 0.120387098 | 0.01732 |
| Roseburia intestinalis | Decreased | 0.063255742 | 0.083731918 | 0.00831 |
| Staphylococcus warneri | Decreased | 0.075342083 | 0.111490793 | 0.01681 |
| Flavonifractor plautii | Decreased | 0.203068198 | 0.257587617 | 0.01099 |
| Pantoea agglomerans | Decreased | 0.544577337 | 0.700982404 | 0.00817 |
| Fusobacterium necrophorum | Decreased | 0.011571322 | 0.206073703 | 0.00435 |
| Oscillibacter valericigenes | Decreased | 0.150652541 | 0.212122148 | 0.01727 |
| Bacillus senegalensis | Decreased | 0.036386936 | 0.063029631 | 0.01091 |
| Prevotella maculosa | Decreased | 0.009832398 | 0.035293692 | 0.00016 |
| Bifidobacterium pseudolongum | Decreased | 0.179676331 | 0.243411188 | 0.03903 |
| Microbacterium gubbeenense | Decreased | 0.075243686 | 0.104802979 | 0.01384 |
| Bifidobacterium cuniculi | Decreased | 0.059159426 | 0.08545889 | 0.0128 |
| Scardovia inopinata | Decreased | 0.056687321 | 0.083842437 | 0.00372 |
| Haemophilus parainfluenzae | Decreased | 0.124007644 | 0.184848973 | 0.00769 |
| Yokenella regensburgei | Decreased | 0.276464005 | 0.384147381 | 0.00277 |
| Lactobacillus malefermentans | Decreased | 0.0193054 | 0.047254935 | 0.00234 |
| Paraprevotella xylaniphila | Decreased | 0 | 0.007558795 | 0.02696 |
| Bacillus thermoamylovorans | Decreased | 0.162180826 | 0.528263319 | 0.0016 |
| Ruminococcus callidus | Decreased | 0.029128125 | 0.07093551 | 0.02268 |
| Bacteroides salyersiae | Decreased | 0.058737348 | 0.078068957 | 0.01169 |
| Sphingobacterium faecium | Decreased | 0.018869721 | 0.040204346 | 0.00256 |
| Anaerococcus obesiensis | Decreased | 0.050870015 | 0.070608219 | 0.01405 |
| Faecalibacterium prausnitzii | Decreased | 0.229985243 | 0.294475715 | 0.0397 |
| Neisseria gonorrhoeae | Decreased | 0.051205583 | 0.093822741 | 8.00E-05 |
| Bacteroides uniformis | Decreased | 0.141582382 | 0.283430255 | 0.00024 |
| Bacteroides vulgatus | Decreased | 1.075364557 | 1.789596826 | 0.03714 |
| Blautia hydrogenotrophica | Decreased | 0.080315408 | 0.166151115 | 0.00066 |
| Clostridium akagii | Decreased | 0.031533172 | 0.050921715 | 0.01603 |
| Rubinisphaera brasiliensis | Decreased | 0.043207338 | 0.060079327 | 0.03932 |
| Fusobacterium naviforme | Decreased | 0.059536737 | 0.095289032 | 0.00369 |
| Paenibacillus glucanolyticus | Decreased | 0.000387229 | 0.038991919 | 0.03673 |
| Tyzzerella nexilis | Decreased | 0.050569844 | 0.068993008 | 0.01331 |
| Helicobacter cinaedi | Decreased | 0.07232658 | 0.119829952 | 0.0173 |
| Moellerella wisconsensis | Decreased | 0.147065594 | 0.462697244 | 0.02097 |
| Clostridium collagenovorans | Decreased | 0.043077106 | 0.078204149 | 0.00224 |
| Lactobacillus rogosae | Decreased | 0.091782676 | 0.175190857 | 0.00198 |
| Janibacter indicus | Decreased | 0.124366605 | 0.164239301 | 0.01956 |
| Blautia faecis | Decreased | 0.058369404 | 0.085490427 | 0.00461 |
| Streptococcus plurextorum | Decreased | 0.020876483 | 0.051167561 | 0.00119 |
| Roseburia faecis | Decreased | 1.105710288 | 1.616837688 | 0.00052 |
| Eubacterium ramulus | Decreased | 0.01873461 | 0.097360508 | 0 |
| Aureimonas altamirensis | Decreased | 0.095578228 | 0.144145343 | 0.0409 |
| Corynebacterium sundsvallense | Decreased | 0.020005011 | 0.03719069 | 0.00108 |
| Sarcina ventriculi | Decreased | 0.180919444 | 0.260142794 | 0.00453 |
| Edwardsiella tarda | Decreased | 0.087559743 | 0.098144645 | 0.00439 |
| Roseburia inulinivorans | Decreased | 0.140026974 | 0.164708832 | 0.01638 |
| Brevibacterium linens | Decreased | 0.036503592 | 0.054968074 | 0.03102 |
| Lachnospira multipara | Decreased | 0.125692951 | 0.188664269 | 0.00893 |
| Ethanoligenens harbinense | Decreased | 0.093113841 | 0.127754813 | 0.02526 |
| Ureaplasma urealyticum | Decreased | 0 | 0.003794365 | 0.02696 |
| Tatumella ptyseos | Decreased | 0.050867438 | 0.095896694 | 0.00023 |
| Blautia stercoris | Decreased | 0.100832844 | 0.123311456 | 0.04197 |
| Enterobacter cloacae | Decreased | 0.508066605 | 0.621144375 | 0.01068 |
| Parasutterella excrementihominis | Decreased | 0.095623103 | 0.143374729 | 0.00814 |
| Ornithobacterium rhinotracheale | Decreased | 0 | 0.005281337 | 0.02696 |
| Odoribacter laneus | Decreased | 0 | 0.005689909 | 0.02696 |
| Acinetobacter calcoaceticus | Decreased | 0.051332031 | 0.093018621 | 0.00016 |
| Cellulosilyticum lentocellum | Decreased | 0.112851227 | 0.176923961 | 0.00565 |
| Corynebacterium freneyi | Decreased | 0.271975421 | 0.356715755 | 0.02097 |
| Lactobacillus mudanjiangensis | Decreased | 0.014540944 | 0.032555208 | 0.0057 |
| Pediococcus pentosaceus | Decreased | 0.076485711 | 0.093159462 | 0.00192 |
| Lactobacillus reuteri | Decreased | 0.023076101 | 0.052112982 | 0.00154 |
| Paraeggerthella hongkongensis | Decreased | 0.092037642 | 0.15060427 | 0.00019 |
| Leptothrix discophora | Decreased | 0.346034659 | 0.564608527 | 6.00E-05 |
| Clostridium sartagoforme | Decreased | 0.098291139 | 0.121572995 | 0.03445 |
| Moraxella lacunata | Decreased | 0.07308659 | 0.110747599 | 0.0287 |
| Mannheimia granulomatis | Decreased | 0.049496305 | 0.101383337 | 0.01965 |
| Hydrogenoanaerobacterium saccharovorans | Decreased | 0.264267725 | 0.367175054 | 0.0183 |
| Streptococcus ictaluri | Decreased | 0 | 0.002348655 | 0.02696 |
| Bifidobacterium angulatum | Decreased | 0.061383406 | 0.085631329 | 0.02074 |
| Achromobacter denitrificans | Decreased | 0.175134956 | 0.202075587 | 0.03192 |
| Bacteroides ovatus | Decreased | 0.063410776 | 0.106402698 | 0.00589 |
| Streptococcus porcinus | Decreased | 0.031423134 | 0.056840597 | 0.00457 |
| Anaerostipes hadrus | Decreased | 0.120870447 | 0.18587972 | 0.00483 |
| Prevotella aurantiaca | Decreased | 0.040594847 | 0.077123046 | 0.00052 |
| Bergeyella zoohelcum | Decreased | 0.017923569 | 0.036337344 | 0.01266 |
| Prevotella paludivivens | Increased | 0.300185351 | 0.150961737 | 0.04413 |
| Aggregatibacter actinomycetemcomitans | Increased | 0.08490779 | 0.063770845 | 0.03033 |
| Brevibacterium casei | Increased | 0.156039256 | 0.073376948 | 0.00022 |
| Propionimicrobium lymphophilum | Increased | 0.550448048 | 0.40415277 | 0.04745 |
| Microbacterium luteolum | Increased | 1.534567203 | 0.878377613 | 0.0066 |
| Cardiobacterium hominis | Increased | 0.113949731 | 0.080642073 | 0.00101 |
| Corynebacterium ulcerans | Increased | 0.091266654 | 0.054629775 | 0.00242 |
| Paenibacillus riograndensis | Increased | 0.246025061 | 0.168368194 | 0.03623 |
| Acidovorax sp | Increased | 0.03845149 | 0.011459504 | 0.00465 |
| Facklamia tabacinasalis | Increased | 1.027116116 | 0.754767337 | 0.03275 |
| Salmonella enterica | Increased | 0.385431803 | 0.0919443 | 0.01577 |
| Methanobrevibacter smithii | Increased | 0.123755026 | 0.083424063 | 0.0196 |
| Bacteroides clarus | Increased | 0.508414271 | 0.161024719 | 0.01127 |
| Brevibacterium epidermidis | Increased | 0.048096026 | 0.019624674 | 0.00277 |
| Bacillus polyfermenticus | Increased | 0.239505774 | 0.110533342 | 0.00483 |
| Blautia caecimuris | Increased | 0.065214 | 0.033797693 | 0.03774 |
| Mobiluncus curtisii | Increased | 0.121820736 | 0.097459527 | 0.0083 |
| Erysipelatoclostridium ramosum | Increased | 0.099220251 | 0.061246113 | 0.00894 |
| Schwartzia succinivorans | Increased | 0.069269187 | 0.019681171 | 0.02311 |
| Brevibacillus brevis | Increased | 0.053826902 | 0.032444017 | 0.01022 |
| Brevibacterium pityocampae | Increased | 0.460792791 | 0.174078477 | 0.01064 |
| Streptococcus mitis | Increased | 0.024249815 | 0.000970346 | 0.03941 |
| Lactobacillus vaginalis | Increased | 0.021237808 | 0.001394156 | 0.02374 |
| Campylobacter coli | Increased | 0.327878653 | 0.194781956 | 0.00022 |
| Kocuria indica | Increased | 0.044340807 | 0.016742598 | 0.00382 |
| Microbacterium paraoxydans | Increased | 0.154878243 | 0.119481404 | 0.00108 |
| Brevundimonas terrae | Increased | 0.260816674 | 0.157609598 | 0.00292 |
| Parabacteroides goldsteinii | Increased | 0.833520344 | 0.506568505 | 0.00336 |
| Bacteroides faecis | Increased | 0.080003485 | 0.046257357 | 0.00141 |
